# Nucleosome Structures Built from Highly Divergent Histones: Parasites and Giant DNA Viruses

**DOI:** 10.3390/epigenomes6030022

**Published:** 2022-08-02

**Authors:** Shoko Sato, Mariko Dacher, Hitoshi Kurumizaka

**Affiliations:** Laboratory of Chromatin Structure and Function, Institute for Quantitative Biosciences, The University of Tokyo, 1-1-1 Yayoi, Bunkyo-ku, Tokyo 113-0032, Japan; satosho@iqb.u-tokyo.ac.jp (S.S.); m_dacher@iqb.u-tokyo.ac.jp (M.D.)

**Keywords:** nucleosome, chromatin, parasite, DNA virus

## Abstract

In eukaryotes, genomic DNA is bound with histone proteins and packaged into chromatin. The nucleosome, a fundamental unit of chromatin, regulates the accessibility of DNA to enzymes involved in gene regulation. During the past few years, structural analyses of chromatin architectures have been limited to evolutionarily related organisms. The amino acid sequences of histone proteins are highly conserved from humans to yeasts, but are divergent in the deeply branching protozoan groups, including human parasites that are directly related to human health. Certain large DNA viruses, as well as archaeal organisms, contain distant homologs of eukaryotic histone proteins. The divergent sequences give rise to unique and distinct nucleosome architectures, although the fundamental principles of histone folding and DNA contact are highly conserved. In this article, we review the structures and biophysical properties of nucleosomes containing histones from the human parasites *Giardia lamblia* and *Leishmania major*, and histone-like proteins from the *Marseilleviridae* amoeba virus family. The presented data confirm the sharing of the overall DNA compaction system among evolutionally distant species and clarify the deviations from the species-specific nature of the nucleosome.

## 1. Introduction

In eukaryotes, highly negatively charged DNA binds to positively charged histone proteins, thereby packaging genomic DNA within the nucleus. The packing architecture, called chromatin, forms a fibrous “beads-on-a-string” structure, in which linker DNAs connect nucleosomes [1]. In the nucleosome, DNA is packed as a spheroid particle with a packing ratio (DNA length: particle diameter) of about 6:1. The nucleosome is a fundamental unit of chromatin, in which 145–147 base pairs of DNA are wrapped around the histone octamer, composed of two each of the four core histone proteins, H2A, H2B, H3, and H4 [2]. This primary repeating unit folds into a higher-order structure with the association of chromatin-related proteins, such as the linker histone H1, and accordingly, the access of enzymes involved in transcription, replication, repair, and recombination to DNA is basically restricted. To achieve these enzymatic processes, chromatin structures are rearranged through alterations of the nucleosome structures, including DNA unwrapping, sub-nucleosome formation, and DNA register changes, facilitated by intrinsic or ATP-dependent remodeling factors [3,4]. The nucleosome surface also serves as a scaffold for chromatin regulatory proteins, with some occasionally performing enzymatic histone modifications [5,6]. Therefore, the nucleosome properties ensure the fine-tuning of nuclear processes.

To date, numerous nucleosome structures from various eukaryotic species, such as *Homo sapiens* [7], *Xenopus laevis* [8,9], *Gallus gallus* [10], *Drosophila melanogaster* [11], and yeasts [12,13], have been determined at atomic resolutions by X-ray crystallography and cryo-electron microscopy (cryo-EM). In addition to the canonical type of histones, the structures of many nucleosomes containing histone variants, which are mostly replication-independently incorporated into nucleosomes and function in specific cell lineages, have been elucidated [14,15,16]. These studies have precisely defined the structural deviations linked to the biophysical properties conferred by the different histones, but the primary structural principles are shared by all nucleosomes. Even in *Archaea*, the nucleosome-like particle formed by the self-octamerization of one histone-like protein (HMfB) is similar to the eukaryotic nucleosome, although the archaeal chromatin adopts a unique polymeric architecture [17,18,19].

In the phylogenic tree of eukaryotes, the evolutionary distance between yeasts and humans is shorter than that of the deeply branching protozoan group. Many previous studies of chromatin structures are limited to relatively evolutionarily close model organisms, and thus the chromatin organization of the protozoan group, including human parasites that are directly related to human health, remains unclear. Intriguingly, histones have also been identified among proteins secreted into host cells by multiple protozoan parasites [20,21]. Here, we describe the primary principles involved in histone folding and DNA wrapping in the nucleosome, and new insights into the structures of nucleosomes containing divergent histones. We discuss the similarities and deviations of recently reported nucleosome-like structures from a large DNA virus, the nucleosome structure from a *Giardia* parasite, and a hybrid nucleosome structure containing a *Leishmania* parasite histone protein.

## 2. Structures of Histone Complexes in Nucleosomes

### 2.1. Histone Fold

**Figure 1:**
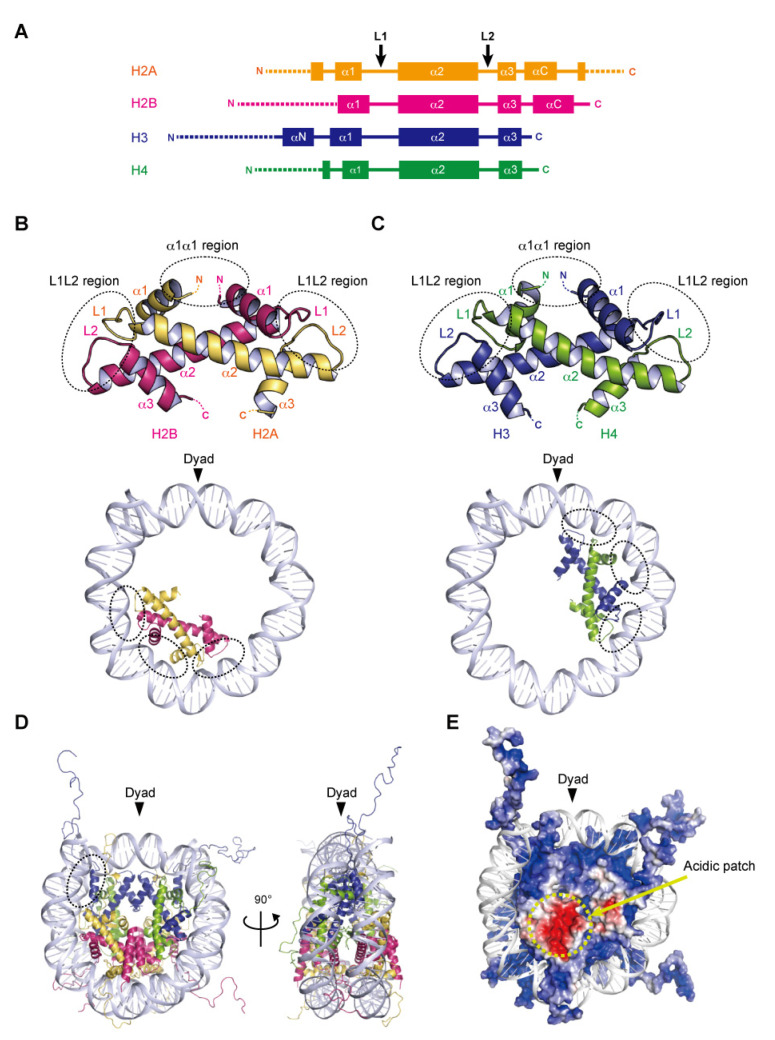
Assembly of the nucleosome. (**A**) Secondary structures of the core histones H2A, H2B, H3, and H4. L1 and L2 show loop 1 and loop 2, respectively. The αN, α1, α2, α3, and αC helices are shown as boxes. The N- and C-terminal tail regions are depicted by dotted lines. (**B**,**C**) Handshake motifs shared by H2A–H2B (**B**) and H3–H4 (**C**) dimers (upper panels). The handshake motifs are shown in approximately one-half of the nucleosomal DNAs (lower panels). Dashed circles show DNA contact sites involving the L1L2 and α1α1 regions. (**D**) Overall structure of the nucleosome core particle. This nucleosome structure was determined by X-ray crystallography at 1.9 angstrom resolution (PDB ID: 1KX5). Dashed circles show a DNA contact site involving the αN helix of H3. (**E**) Electrostatic potential surface of the histone octamer, represented by negative and positive charges in red and blue, respectively.

Nucleosome formation is similar from humans to yeast, and even to *Archaea*, as the basic structures of the folded histones are shared in various species. In the nucleosome, the central ‘disc’ architecture is composed of four core histones, H2A, H2B, H3, and H4, sharing well-conserved motifs termed histone-fold domains comprising three α-helices, α1, α2, and α3, separated by two loops, L1 and L2 [22,23] (Figure 1A). The H2A–H2B and H3–H4 heterodimers are assembled into the characteristic handshake motif via central hydrophobic interactions, along with electrostatic contacts and hydrogen bonds [8] (Figure 1B,C). Two H3–H4 dimers are joined into a (H3–H4)_2_ tetramer in physiological ionic strength solutions by the interactions of the C-terminal halves of the α2 helices of H3 and H3′ (adjacent copy of H3) and the α3 helix of H3. A single H3–H4 tetramer and two H2A–H2B dimers are incorporated into the nucleosome core particle to form the histone octamer, around which the double-stranded DNA is wrapped (Figure 1D). Within the octamer, the H2A–H2B dimers are assembled by the interaction of H2B with H4, in addition to the H3–H3′ interaction. These remarkable associations are described as the H2B–H4 four-helix bundle and the H3–H3′ four-helix bundle, respectively [8]. 

### 2.2. DNA Wrapping around the Histone Octamer

In the nucleosome, 145–147 base pairs of DNA are wrapped around the histone octamer in 1.65 left-handed superhelical turns with 14 contact sites on the histone surface, where the DNA phosphate backbone faces the histone octamer. Twelve of the 14 DNA–histone contact sites are mediated by the handshake motifs, which are divided into two types: the α1α1 and L1L2 regions that are enriched in positively charged residues (Figure 1B,C). The α1α1 region is formed by the N-terminal ends of the α1 helices of the histone heterodimer pair (Figure 1B,C). The L1L2 region is formed by the L1 and L2 loops and the C-termini of the α2 helices on each end of the heterodimer pair (Figure 1B,C). A total of four α1α1 and eight L1L2 regions interact directly with 12 helical turns (120 bp) of the DNA in the minor grooves. The two remaining DNA–histone contact sites originate from the N-terminal α helix (αN) and the N-terminal extension of H3, where the positively charged residues bind to the DNA at the entry–exit sites of the nucleosome (Figure 1D). Related to the studies of histone variants, some mutational analyses have clearly shown the contributions of arginine residues, at positions 42 [24], 49, and 53 [25] on the αN and N-terminal extension of H3, to the DNA flexibility at the entry–exit sites. Additionally, the interaction of the C-terminal region of H2A with the H3–H4 tetramer guides this last turn of the nucleosomal DNA (Figure 1D). The histone variant H2A.B (formerly H2A.bbd) lacking the C-terminal region forms a nucleosome with ~110 base pairs of DNA wrapped around the octamer [26,27]. The flexibility of the DNA at the entry–exit sites also plays a considerable role in the higher-order structure of chromatin.

### 2.3. Acidic Patch of the Nucleosome

In many eukaryotic nucleosomes, the acidic patch is a fundamental and well-conserved region located on the disc surface. The acidic side chains of amino acid residues in the α2 helix of H2A (Glu56, Glu61, Glu64, Asp90, Glu91, and Glu92 in human) and the αC helix of H2B (Glu105 and Glu113 in human) form a negatively charged patch exposed to the solvent (Figure 1E). The acidic patch acts as a binding platform for many proteins that regulate chromatin function via arginine anchoring, including histone modification enzymes, chromatin remodelers, and DNA methyltransferases [6,28]. In addition, X-ray crystallographic analyses have shown that the basic residues in the N-terminal tail of H4 contact the acidic patch, in a manner reminiscent of the nucleosome–nucleosome interactions in nucleosome arrays, suggesting that the acidic patch may also be involved in chromatin compaction [28,29].

### 2.4. Histone Tails

Histone proteins contain intrinsically disordered regions at the N-termini of H2A, H2B, H3, and H4, and the C-terminus of H2A (Figure 1A,E). These flexible ‘tails’, which protrude from the central disc architecture of the nucleosome core, are partly exposed to the solvent. With their many positively charged residues, such as lysine and arginine, the histone tails associate with the negatively charged DNA in the nucleosome core particle or linker regions [30,31]. However, the histone tails do not seem to affect the disc architecture of the nucleosome, as suggested from the X-ray structures of N-terminally truncated histones [32]. In contrast, the histone tails likely alter the higher-order structure of chromatin through the interactions between the H4 tail and the acidic patch [33]. Biophysical evidence has indicated the contribution of histone tails to the compaction of nucleosome arrays [34]. Importantly, the histone tails are often enzymatically modified in vivo, by the acetylation, methylation, and ubiquitylation of lysine residues, and the phosphorylation of serine and threonine residues [35]. These post-translational modifications serve as binding sites for factors that regulate the chromatin structure and function [5,36,37]. Although the histone tail sequences are relatively diverse among eukaryotic species, the residues subjected to post-transcriptional modifications are highly conserved. In particular, the H3 Lys4, Lys9, Lys27, Lys36, and H4 Lys20 residues, which are targeted for methylation and acetylation, are inferred to be conserved lysine residues even in the last eukaryotic common ancestor (LECA) [38].

## 3. Structures of Nucleosomes Containing Parasite Histones

### 3.1. Genomic Organization and Gene Regulation in Giardia

*Giardia lamblia* is a flagellated unicellular eukaryote that parasitizes the small intestines of humans and animals. *Giardia* causes a diarrheal disease termed giardiasis, which is one of the most common parasitic infections throughout the world [39,40]. The *Giardia* life cycle consists of two main forms: the non-infectious trophozoite that causes the primary symptoms of diarrhea by proliferating on the surfaces of the host intestinal cells, and the highly infective cyst form, which is environmentally resistant and can survive outside the host. This parasite is classified in the order *diplomonadia* within the *Excavata* supergroup. In the eukaryotic tree of life, *Giardia* is in a deeply branched position, with a simplification of most cellular processes, and lacking certain organelles, such as mitochondria and peroxisomes [41]. *Giardia* has two nuclei with equivalent activities. Consistent with higher eukaryotes, genomic regulation occurs on the “beads-on-a-string” chromatin structure [42]. The 12 mega base pairs of the *Giardia* genome contain 4963 open reading flames with few introns, short intergenic and 3′ and 5′ untranslated regions, and small promoter regions that are close to transcriptional start sites [43,44,45]. *Giardia* has a simple transcriptional initiation complex [43], with few transcriptional factors [46] and chromatin remodeler subunits [47]. A genome-wide transcriptional analysis indicated that bidirectional transcription produces both sense and antisense transcripts in *Giardia* [48]. In addition, *Giardia* possesses an RNA interference system, which is involved in antigenic switching, conferring a pathogenic ability to evade the host immune system [49]. These results suggest that transcriptional control is limited in *Giardia*. In the encystation process, the transformation from trophozoite to cyst stage, histone modifications such as ubiquitylation, deacetylation, and methylation have been verified, indicating that the chromatin-based regulation of gene expression is essential for biological processes in *Giardia* [50,51,52,53,54].

### 3.2. Structure of the Nucleosome Containing Giardia Histones

The *Giardia* genome encodes two copies of the H2A, H2B, and H3 genes, and three copies of the H4 genes for canonical histones, and their corresponding mRNAs are polyadenylated [44,55]. Histone variants H3B and CenH3 localized in the centromere were identified, but not the linker histone H1 [56]. In higher eukaryotes, the phosphorylation of an H2A variant, the H2A.X Ser139 residue (γH2A.X), is responsible for facilitating DNA repair in response to double-stranded breaks [15,36,57]. Although no H2A variant has been identified in *Giardia*, the “Ser-Gln-Asp-Leu” motif, within the H2A.X variant in higher eukaryotes, is present in the H2A C-terminus. The four core histones share the typical eukaryotic features of histone-fold domains, containing three α helices separated by two loops [55]. Parasite histones generally have diverse amino acid sequences, with remarkably low sequence identity compared to metazoan histones [58,59]. Indeed, the identities of the *Giardia lamblia* (*G. lamblia*) histone-fold domains of H2A, H2B, H3, and H4 compared to the human histone-fold domains are 48, 49, 60, and 78%, respectively. As in other eukaryotes, the *Giardia* core histones contain intrinsically disordered “tail” regions that undergo conserved post-translational modifications. Emery-Corbin and colleagues identified *Giardia* histone modifications, including methylation, acetylation, and phosphorylation, using mass spectrometry [60]. They demonstrated that *Giardia* histone tails are highly modified (more than 50 sites were identified) and that these histone modifications in *Giardia* are largely equivalent to those in many other eukaryotes, suggesting that similar epigenetic mechanisms exist in this parasite [60]. Among the well-conserved modifications, the phosphorylation of H3 Ser10, and the methylations of H3 Lys4, Lys36, Lys9 and Lys27, but not H3 Lys79 and H4 Lys20, were identified in *Giardia*.

**Figure 2:**
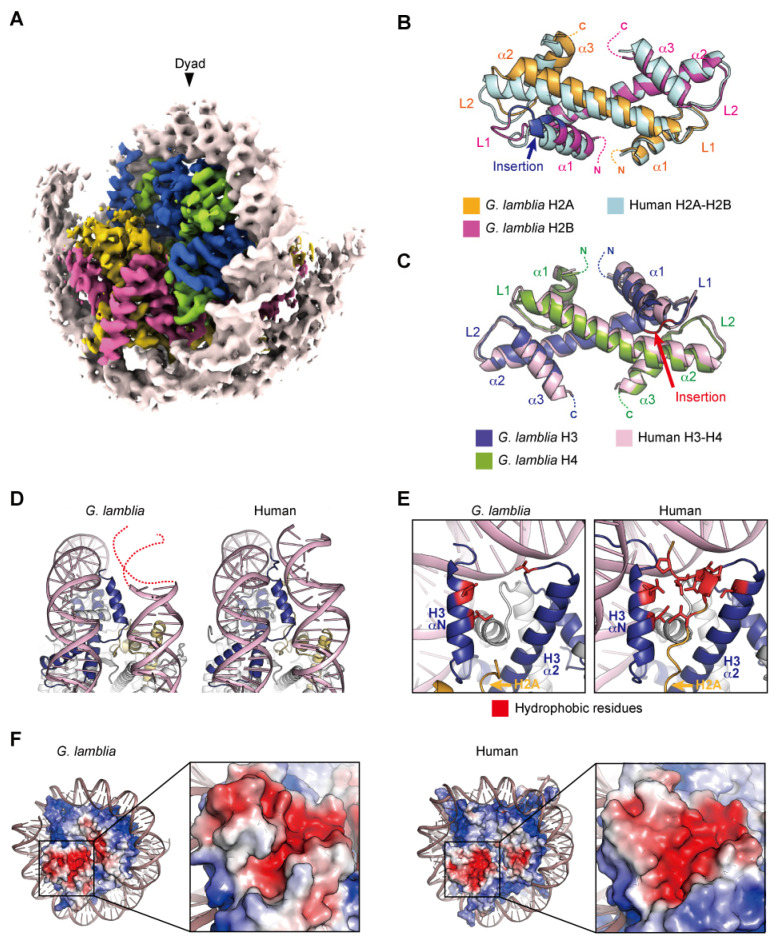
Nucleosome structure containing *G. lamblia* core histones. (**A**) Overall structure of the *G. lamblia* nucleosome (PDB ID: 7D69). (**B**,**C**) Handshake motifs of H2A–H2B (**B**) and H3–H4 (**C**) dimers in the *G. lamblia* nucleosome are superimposed with H2A–H2B and H3–H4 dimers in the human nucleosome (PDB ID: 6R93), respectively. (**D**) Entry–exit DNA regions of the *G. lamblia* nucleosome and the corresponding regions in the human nucleosome. Dashed lines represent predicted DNA. (**E**) Close-up views of the DNA and histones near the entry–exit DNA regions of *G. lamblia* and human nucleosomes with the H3 αN and α2 helices and L2 regions. The hydrophobic residues of the H2A C-terminal region form a hydrophobic core with the hydrophobic residues of H3, which is only visible in the human nucleosome. (**F**) Electrostatic potential surfaces and close-up views of the acidic patch of the *G. lamblia* and human nucleosomes are shown.

The cryo-EM structure of the *G. lamblia* nucleosome was determined at a 3.6 angstrom resolution, after reconstitution with 145 base pairs of modified Widom 601 (601L) palindromic DNA [61]. The overall structure of the *G. lamblia* nucleosome resembles that of the human nucleosome (Figure 2A). All *G. lamblia* histones fold into the characteristic handshake motifs, but with notable deviations from the main chains of human histones in the nucleosome, including the *G. lamblia*-specific insertions of six and two residues in H2B and H3, respectively (Figure 2B,C). This six-residue insertion extends the α1 helix and L1 loop of *G. lamblia* H2B and alters the conformation of the L1L2 region (Figure 2B). Importantly, the insertion affects the shape and peptide-binding properties of the adjacent acidic patch (see below). In the cryo-EM structure of the *G. lamblia* nucleosome, only 125 base pairs of DNA are wrapped asymmetrically around the histones, and the DNA flexibility was confirmed in solution (Figure 2A). Furthermore, in the cryo-EM structure of the *G. lamblia* nucleosome, only one of the shortened αN helices of H3 was resolved, and the two C-terminal regions of H2A were not visualized (Figure 2D,E). In the human canonical nucleosome, the αN helix and N-terminal extension of H3 bind directly to the last turn of the superhelical DNA, and these interactions are considered to be guided by the C-terminal region of H2A via a hydrophobic cluster. In *G. lamblia*, the hydrophobic residues of the H2A C-terminal region are replaced by hydrophilic or small aliphatic residues. A mutational analysis revealed that the C-terminal region of *G. lamblia* H2A is responsible for the flexibility of the DNA entry–exit sites in the nucleosome. Consistent with this DNA flexibility, the nucleosome array containing *G. lamblia* histones appears to adopt a more relaxed conformation, compared to the human nucleosome array. In addition, a biological analysis revealed the instability of the *G. lamblia* nucleosome. These properties of relaxed chromatin and nucleosome instability could facilitate transcriptional activation in *Giardia*, which possesses simple gene regulatory systems.

In general, the H2A α2 and H2B αC helices contain acidic residues and thus form the well-conserved acidic patch on the nucleosome surface. In the *G. lamblia* nucleosome, the acidic patch is formed by the residues Glu53, Glu58, Glu62, Glu87, and Asp89 of H2A, and Glu104 and Glu112 of H2B. However, the shape of the acidic patch in the *G. lamblia* nucleosome is substantially different from that in the human nucleosome, due to a ridge formed by the H2B insertion that deepens the acidic patch (Figure 2F). Indeed, the *G. lamblia* nucleosome was defective in binding to the LANA peptide, a viral peptide that binds efficiently to the acidic patch in the human nucleosome [29,62]. The functions of the acidic patch and the H2B insertion in *Giardia* cells are unknown. A comprehensive analysis revealed the phosphorylations of H2B Ser45 in the six-residue insertion and the adjacent Thr49, which are unique residues in *Giardia* [60], although their functions remain unknown. In addition, some counterparts of acidic patch recognition proteins, including Dot1L, SET8, and RCC1, have not been identified [38]. Chromatin regulatory proteins that recognize the *Giardia* acidic patch or the H3 L1 loop could have unique structures and become potential targets for drug development.

### 3.3. Genome and Histone Proteins of Leishmania

The protozoan parasites in the genus *Leishmania*, in the family *Trypanosomatidae,* are the causative agents of severe immunopathologies known as leishmaniases [63]. *Leishmania* infections can affect mucosal and cutaneous surfaces, as well as several internal organs, the latter of which can lead to the death of the infected host if left untreated [64]. Leishmaniasis is a serious public health problem worldwide, with one billion people living at risk of infection [63]. After transmission to humans and animals by a blood-feeding sandfly bite, the parasite invades macrophages and immediately deploys its phenomenal ability to colonize and subvert immune cells to ensure its survival in the host cell.

The 32.8 mega base pairs of the *Leishmania* genome contain clusters of large polycistronic gene units, corresponding to up to hundreds of genes arranged on the same DNA strand. These clustered genes are transcribed into long polycistronic pre-messenger RNAs that will be processed by trans-splicing, as in other trypanosomatids [65,66,67]. In addition, most of the genes lack introns and conventional promoters [68,69]. Trypanosomatids are biologically interesting because their chromatin remains decondensed as fine fibers, instead of condensing into a chromosome, during cell division [70]. However, the DNA of these parasites is packed in nucleosomes, as in higher eukaryotes [71,72,73,74]. In *Leishmania*, each of the core histones, H2A, H2B, H3, and H4, is encoded by multiple genes [75,76,77,78]. *Leishmania* histones share 48–60% identity with human histones, showing their evolutionary diversification [79]. The *Leishmania* genome also encodes the linker histone H1 [79] and three histone variants, the essential H2A.Z and H2B.V, and the non-essential H3.V [79,80].

Many proteomic studies have demonstrated the release of virulence factors by various protozoan parasites into the host cell environment, suggesting that these proteins could play an important role for parasite survival in the host [20,21,81,82,83,84,85,86,87,88,89,90,91]. Several studies have shown that *Leishmania* modifies the epigenetic state of the host cell upon invasion, but little is known about the virulence factors that regulate this subversion mechanism [92,93,94,95,96]. Interestingly, the four histones H2A, H2B, H3, and H4, and a nucleosome assembly protein (NAP1)-like protein have been identified among the secreted proteins in *Leishmania,* suggesting potential epigenetic-related mechanisms by which the parasite could subvert its host cell [20,82,83,84]. Ectopically produced *Leishmania major* H3 (LmaH3), which mimics LmaH3 released in infected cells, is incorporated into human chromatin in HeLa cells [97]. In addition, LmaH3 was incorporated into the mononucleosomal fractions of chromatin isolated from HeLa cells that stably expressed LmaH3 fused to GFP, and these results were also validated by FRAP experiments using living HeLa cells [97]. This is the first study showing direct interactions between the parasite histones and host histones as part of their nucleosomes.

### 3.4. Nature of the Parasitic–Human Hybrid Nucleosome

**Figure 3:**
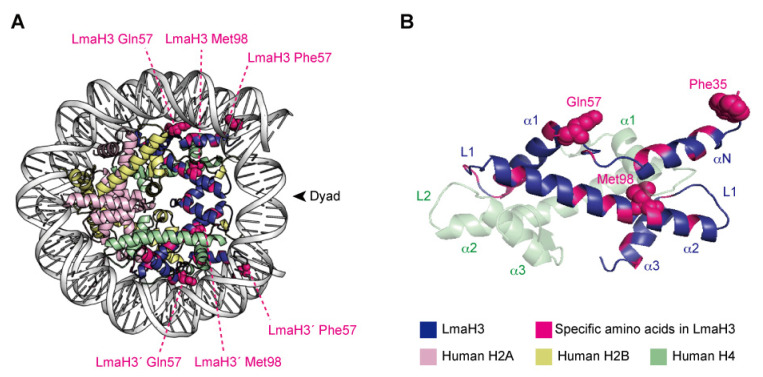
The hybrid nucleosome structure containing *L. major* H3 (LmaH3) and human H2A, H2B, and H4. (**A**) Overall structure of the hybrid nucleosome (PDB ID: 6KXV). (**B**) The LmaH3 and human H4 dimer structures in the hybrid nucleosome.

X-ray crystallographic analyses demonstrated that LmaH3 forms a conventional nucleosome structure with human histones H2A, H2B, and H4, using the palindromic human α-satellite 146 bp DNA in vitro [97] (Figure 3A). As for the biochemical and biophysical properties, the authors found that the LmaH3 nucleosome exhibits reduced stability and relaxed chromatin conformation compared to the human nucleosome. In *Leishmania* H3, the amino acid residues Trp35 and Gln57 replace the respective human H3 Tyr41 and Arg63 residues, which interact with DNA (Figure 3B). The Phe104 residue of human H3.1, located at the H3–H4 interface, is replaced by Met98 in *Leishmania* H3 (Figure 3B). Mutation analyses revealed that the residues Tyr41 and Arg63, and especially Phe104 of H3, hinder the association of H3–H4 with DNA, drastically decreasing the stability of the H3–H4–DNA complex. The nucleosome instability and relaxed chromatin folding induced by the incorporation of parasitic histones may impact the higher-order chromatin configuration of infected cells, resulting in altered epigenetic states and gene expression patterns, to favor the establishment and survival of the *Leishmania* parasite. This study provided more evidence that *Leishmania* may hijack the host’s epigenetic status maintained by the chromatin structure and dynamics during infection. The secretion of histones and other epigenetic proteins appears to be a widespread phenomenon in pathogenic parasites. The other four histones have also been detected as secreted proteins in various parasites such as *Trypanosoma* [21,87] and *Plasmodium* [81,88], as well as fungal pathogens such as *Histoplasma* and *Cryptococcus* [89,90], and the helminthic parasitic *Echinococcus* [91]. We can therefore speculate that some parasites may use stratagems targeting host chromatin by exploiting their secreted chromatin proteins, including the parasitic–human hybrid nucleosome strategy, to maximize their survival in the host.

## 4. Nucleosome-like Structure Containing Viral Histone Doublets

**Figure 4:**
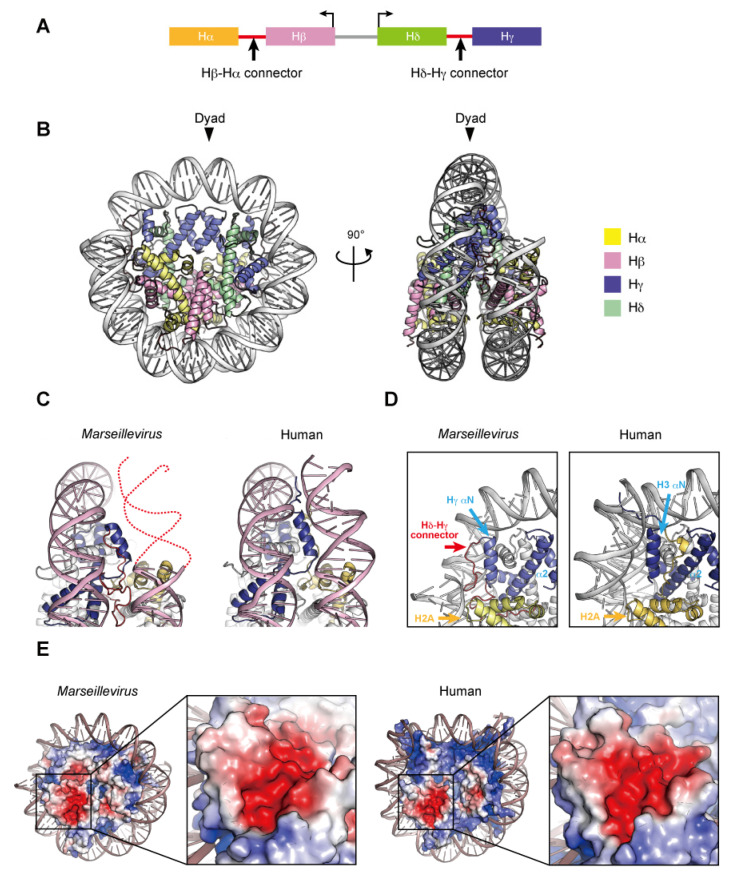
Nucleosome structure containing *Marseillevirus marseillevirus* core histones. (**A**) Organization of the viral histone doublets. (**B**) Overall structure of the *Marseillevirus* nucleosome (PDB ID: 7LV8). (**C**) Entry–exit DNA region of the *Marseillevirus* nucleosome and the corresponding region in the human nucleosome (PDB ID: 5AV9). Dashed lines represent predicted DNA. (**D**) Close-up views of the DNA and histones near the entry–exit DNA regions of *Marseillevirus* nucleosomes with the Hγ αN and α2 helices (left panel), Hδ–Hγ connector (left panel), and the corresponding region in the human nucleosome (right panel). The H2A C-terminal region of H2A is only visible in the human nucleosome. (**E**) Electrostatic potential of the histone octamers and close-up views of the acidic patch. The equivalent regions of the *Marseillevirus* and human nucleosomes are shown.

Two nucleosome-like structures from related large DNA viruses have been reported [98,99]. In 2003, the first giant virus was discovered in a eukaryotic Amoebozoa named *Mimivirus*, a nucleocytoplasmic large DNA virus (NCLDV) with a 1.2 mega base pair DNA genome encoding approximately 1200 open reading frames [100,101]. *Marseilleviridae*, including *Melbournevirus* and *Marseillevirus*, is an established family among the large amoebal NCLDVs, which have icosahedral shapes with a diameter of ~250 nm, and double-stranded circular DNA genomes that are more than 300 kilo base pairs in length [102]. The NCLDV species appear to share a core set of conserved genes, including the translation machinery [103], DNA polymerase subunits [102], an RNA polymerase subunit [104], and other common enzymes. To date, genes encoding histone-like proteins have been identified in many giant viral genomes, albeit some viruses have only one histone gene [105]. Four core histone homologs are encoded in *Marseilleviridae* genomes [106,107]. The *Marseilleviridae* histone proteins, Hα, Hβ, Hγ, and Hδ, correspond to H2A, H2B, H3, and H4, respectively, based on the homology of their histone-fold domains. The N-termini of Hα and Hγ fuse with the C-termini of Hβ and Hδ, respectively, through a connector, and these Hβ–Hα and Hδ–Hγ fusions are described as histone doublets (Figure 4A). Phylogenic analyses suggested that the histone doublets of *Marseilleviridae* were possibly acquired from eukaryotic histones, prior to the divergence of histone variants such as H2A.Z and CenH3 at the proto-eukaryotic stage of evolution, and thus are different from those encoded in archaeal genomes [108].

The histone doublets from two giant viruses can form nucleosome-like structures (described here as virus nucleosomes) in vitro. Velencia-Sanchez and Abini-Agbomson et al. determined the cryo-EM structure of the nucleosome containing histone doublets of *Marseillevirus marseillevirus* assembled with 147 bp of Widom 601 DNA [98]. Liu et al. also elucidated the cryo-EM structures of *Melbournevirus* nucleosomes reconstituted with 147 and 207 bp of Widom 601 DNA [99]. These structures revealed that the viral histone doublets associated with DNA form an architecture remarkably similar to that of the eukaryotic nucleosome (Figure 4B). In the viral nucleosomes, the two histone domains of each doublet adopt the handshake motif and then assemble into a heterotetramer through the four-helix bundles of Hγ–Hγ′ and Hδ–Hβ, corresponding to the four-helix bundles of H3–H3´ and H2B–H4, respectively, in the eukaryotic octamer. In eukaryotes, the H3–H3′ four-helix bundle is primarily established by hydrophobic interactions, the intramolecular H3 Asp123–H3´ Arg116 salt-bridge, and the intermolecular H3 His113–H3 Asp123 interaction [8]. In *Melbournevirus*, the Hγ–Hγ′ four-helix bundle is established by hydrophobic interactions, as in eukaryotes, and by the viral counterpart Hγ Asp204–Hγ′ Arg197 salt-bridge but lacks an intermolecular histidine–aspartic acid interaction, since His113 is substituted by the viral counterpart of Ser194. On the contrary, hydrophobic interactions and salt-bridges are not well conserved in the H2B–H4 four-helix bundle, which is rearranged due to substituted residues [98]. The viral H2β α2 helix is one turn shorter than the eukaryotic sequence [99]. Additionally, the direct interactions between the H2A and H2A′ L1 loops in eukaryotic nucleosomes are lost in the viral Hα counterpart. Lui et al. demonstrated the instability of the *Melbournevirus* nucleosome [99]. These structural alterations may lead to the instability of viral nucleosomes, together with the Hα C-terminal region (see below). 

In viral nucleosome structures, approximately ~120 base pairs of DNA are wrapped around the viral histones, although the main principles of histone–DNA interactions are conserved. In eukaryotes, the H3 αN helix guided by the H2A C-terminal domain contributes toward stabilizing the last turn of the 147 base pair superhelical DNA organized in the nucleosome (Figure 4C). The Hγ αN helix is shorter than the H3 αN helix and lacks the canonical interface with DNA but presents an arginine/glutamine motif on the Hδ–Hγ connector that contacts the DNA. In addition, the Hα C-terminal region, with a divergent sequence from the eukaryotic H2A C-terminal region, is disordered in the *Marseillevirus* nucleosome [98] (Figure 4D) and rearranged toward the dyad axis in the *Melbournevirus* nucleosome [99]. 

In eukaryotes, the acidic patch is composed of eight residues in H2A (Glu56, Glu61, Glu64, Asp90, Glu91, and Glu92) and H2B (Glu105 and Glu113). In viral nucleosomes, six residues in Hα (Glu150, Glu155, Glu158 and Asp184) and Hβ (Asp84 and Glu92) form the acidic patch, resulting in size and charge reductions, compared to eukaryotic canonical nucleosomes (Figure 4E). The acidic patch may also serve as a ‘hot spot’ recognized by regulatory proteins in viruses.

Liu et al. detected the localization of the virus histone doublets in host cells. Transfected GFP-fused *Melbournevirus* histone doublets accumulated in the host amoeba cytoplasm after infection, indicating that viral histone doublets are recruited for viral DNA packaging and virion production in the viral factory [99]. Furthermore, they showed that histone doublet-deficient viruses were excluded from host cells throughout infection cycles and are thus inferior to wild-type viruses [99]. Their results indicated that virus histones are essential for viral infectivity and are mostly associated with viral DNA packaging in the capsid. Bryson et al. assessed whether viral nucleosomes assemble on *Marseillevirus* DNA in virions [109]. They reported that after permeabilization of *Marseillevirus* virions, the viral genome was digested with micrococcal nuclease, which predominantly produced 121 base pair DNA fragments [109]. Based on these results, viral histone doublets assemble their genomic DNA into chromatin in virions [105,109]. The cryo-EM structure lacking one copy of Hβ–Hα similar to eukaryotic hexasome is also observed [98]. These findings suggest the dynamics and/or structural versatility of viral nucleosomes. The viral chromatin may play a role in organizing DNA accessibility and thus help to regulate gene expression. In mammalian cells, a DNA sensor protein, cGAS, captures the infected exogenous DNA and induces inflammation [110]. Interestingly, the exogenous DNA becomes insensitive to cGAS by nucleosome formation, because cGAS is inactivated in the complex with a nucleosome [111,112,113,114,115]. Therefore, the viral nucleosome formation may have a function for escaping from the host immune systems, such as cGAS, although functionally homologous proteins have not been identified in the amoeba cell hosts of the large DNA viruses.

## 5. Conclusions

Over the past three decades, many eukaryotic nucleosome structures have been revealed at atomic resolutions, including novel structures of nucleosomes containing histones from the *G. lamblia* parasite and histone-like proteins from *Marseilleviridae* of the large DNA virus family, both with highly divergent histone sequences. As in the ancestral histone-like proteins of HMfB from *Archaea*, and even viruses, the overall structures are well-conserved in the nucleosome or nucleosome-like particles, in which the DNA is left-handedly wrapped around handshake motifs with predominantly conserved histone–DNA contact sites. It would be quite interesting to determine whether the high degree of histone sequence divergence influences the complex relationship between chromatin structure and gene regulatory mechanisms. In addition, expanding the knowledge of the chromatin structures of parasite species will help fill the knowledge gaps in the chromatin characteristics of related parasites and also reveal new targets for future drug development strategies. A novel function has been proposed for the secreted *L. major* histone H3, which may potentially modify the host chromatin structure by infiltrating the host nucleosome. The secreted histone incorporation may cause aberrations of host chromatin homeostasis and thus change the host cell status to one suitable for parasite life. Therefore, it is exciting to imagine that several chromatin proteins released by *Leishmania*, or other parasites, could also serve as virulence factors by disrupting the host’s epigenetic system, thus actively participating in the infection mechanism. Further studies to clarify the unknown functions of these chromatin proteins to better understand parasite biology are awaited.
